# *Balamuthia* Amebic Encephalitis

**DOI:** 10.3201/eid1008.040139

**Published:** 2004-08

**Keywords:** *Balamuthia*, amebic encephalitis, Hispanic-American ethnicity

**To the Editor:** *Balamuthia mandrillaris*, a free-living soil ameba, can cause granulomatous amebic encephalitis as well as nasopharyngeal, cutaneous, and disseminated infections in humans, nonhuman primates, and other animals. Approximately 100 published and unpublished cases of *Balamuthia* amebic encephalitis (BAE) have been reported; most were fatal. Diagnosis of BAE is usually made at autopsy, and rarely by biopsy, in part because the amebas can be overlooked in histopathologic preparations. In recognizing BAE as a type of encephalitis that might otherwise be undiagnosed, the California Encephalitis Project (1) has been screening selected serum samples from patients with encephalitis for evidence of antibodies to *Balamuthia*.

We describe cases of BAE in California and compare data with national data collected on *Balamuthia* infections since the discovery of the organism in 1990. Since 1998, serum and other samples (cerebrospinal fluid [CSF], throat and rectal swabs, brain tissue) from patients with encephalitis have been submitted to the California Encephalitis Project by participating physicians throughout California. The goal of the California Encephalitis Project is to provide enhanced diagnostic testing for etiologic agents of encephalitis through an intensive testing algorithm. The case definition of encephalitis is encephalopathy, plus one or more of the following: fever, seizures, focal neurologic findings, CSF pleocytosis, or electroencephalographic or neuroimaging findings consistent with encephalitis (1). Persons with HIV/AIDS, severel*y* immunocompromised patients, and patients <6 months of age are excluded from the project.

Serum samples were selected for screening for *Balamuthia* antibodies if the patient had clinical or laboratory features suggestive of *Balamuthia* encephalitis (elevated CSF protein and leukocyte counts or compatible findings on neuroimaging) and a history of outdoor occupational (agriculture or construction work) or recreational (camping or swimming) activities during which they may have been exposed to pathogenic or opportunistic free-living amebas. During the study, 215 (approximately 25%) of the >850 serum samples collected in California were tested for *Balamuthia* infection by indirect immunofluorescence assay (2). Testing was conducted on acute-phase serum and a follow-up sample, when available. Serum samples were tested at dilutions from 1:2 to 1:4,096. Positive and negative control samples were run in parallel, with titers from 1:128 to 1:256 for the former and negative to 1:32 for the latter. Serum samples from patients with *Balamuthia* encephalitis did not cross-react with *Acanthamoeba* or *Naegleria*, two other amebas associated with amebic encephalitis (3).

Three (1.4%) of 215 samples tested were positive for antibodies to *Balamuthia* with titers of 1:128, 1:128, and 1:256. In the course of the study period, serum samples from four additional persons, including serum from one person who had been diagnosed by the Centers for Disease Control and Prevention (CDC), who were not part of California Encephalitis Project were positive. The diagnosis of *Balamuthia* encephalitis was confirmed histologically or by indirect immunofluorescence staining of tissue sections in all seven cases; in one case amebas also were isolated in culture from necrotic brain tissue at autopsy (4). All patients were immunocompetent and of Hispanic American ethnicity, and all died. Case-patients included two adults and three children who were native Californians, a child who had arrived from Mexico the previous year, and a child who was a native of Texas who had been diagnosed by the California Department of Health Services (5). The observation that all were of Hispanic American ethnicity prompted a search through CDC's records (N = 104) to confirm the ethnicity of BAE patients throughout the world (G.S. Visvesvara, unpub. data). Patients were considered to be of Hispanic American ethnicity if they were identified as such in case histories or if they had traditional Hispanic surnames. Specific confirmation of ethnicity was not available in the CDC records, and reliance on surnames to determine ethnicity might be a source of error; some Hispanic American persons may have surnames that are not considered to be ethnically Hispanic, and vice versa. According to the records, approximately 50% of the 50 North American patients, which were confirmed by direct immunofluorescence, histopathology, or both, were Hispanic American. Thirty-six percent of all the BAE cases occurred in Latin America. Eleven cases have occurred in California since the early 1990s, including those described above, and all but two were fatal (6). Eight (73%) of these 11 cases occurred in Hispanic Americans.

BAE is not an insignificant disease in California, with 11 cases and 9 deaths reported in the state in the last decade. By comparison, five deaths from indigenous rabies have been reported in the state since approximately 1990 (7). Furthermore, BAE is likely underdiagnosed because of unfamiliarity with appearance of amebas in tissue sections and nonspecific symptoms. Unless there is a high degree of suspicion, it is unlikely that testing for *Balamuthia* would be conducted. Most cases are diagnosed on autopsy, which is often not allowed by families. Also, BAE develops in a disproportionate number of Hispanic Americans. Hispanic Americans make up 12.5% of the U. S. population (United States Census Bureau statistics for 2000) but represent approximately 50% of the cases of BAE. In California, where Hispanic Americans make up 32% of the state's population, they have 73% of BAE cases (p = 0.001, Fisher exact test). In the California Encephalitis Project, Hispanic Americans accounted for approximately 25% of all cases of encephalitis, 26% of serum samples examined for *Balamuthia* antibody, and 21% of cases of viral and bacterial encephalitis, but all BAE patients (n = 3) were in Hispanic Americans (Figure).

**Figure:**
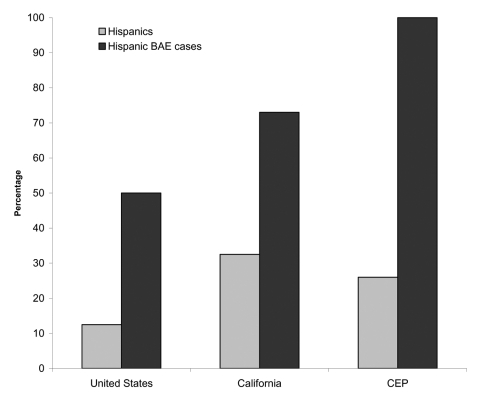
The graph compares Hispanic American populations and Hispanic American *Balamuthia* amebic encephalitis (BAE) cases in the United States, California, and those samples tested for *Balamuthia* antibody in the California Encephalitis Project (CEP). In each of the three groups, the percentage of Hispanic Americans in the population is compared to the percentage of BAE cases in Hispanic Americans.

*Balamuthia* lives in soil (4) and can enter through the respiratory tract or breaks in the skin. Hispanic Americans may be more likely to reside in agrarian settings with increased exposure to soil and opportunities for contamination of cuts and other injuries. Whether caused by environmental factors, genetic predisposition, access to medical care, or other socioeconomic factors and pressures, the reasons for the higher incidence of BAE in Hispanic Americans warrant further study.

This study was supported by the Emerging Infections Program of the CDC.
